# Effectiveness of agalsidase alfa enzyme replacement in Fabry disease: cardiac outcomes after 10 years’ treatment

**DOI:** 10.1186/s13023-015-0338-2

**Published:** 2015-09-29

**Authors:** Christoph Kampmann, Amandine Perrin, Michael Beck

**Affiliations:** Section Head for Congenital Heart Defects, Center for Pediatric and Adolescent Medicine, University Medical Center, University of Mainz, Langenbeckstr. 1, Mainz, DE-55101 Germany; Statistical Programmer, Rare Diseases Business Unit, Global Outcomes Research, Shire, Zug, Switzerland; Professor Emeritus, Department of Pediatrics, University Medical Center, University of Mainz, Mainz, Germany

**Keywords:** Agalsidase alfa, Cardiomyopathy, Enzyme replacement therapy, Fabry disease, Left ventricular hypertrophy, Lysosomal storage disorder

## Abstract

**Background:**

To explore long-term effects of agalsidase alfa on Fabry disease cardiomyopathy in adults.

**Methods:**

Retrospective analysis of prospectively collected data at a single center in Mainz, Germany, revealed that 45 adult patients (21 men, 24 women) had received agalsidase alfa for approximately 10 years. Data were extracted for cardiac and heart failure status, echocardiographic evaluations of cardiac structure and function, and renal function at treatment start and during agalsidase alfa treatment.

**Results:**

After 10 years of agalsidase alfa treatment, heart failure classification had improved by at least 1 class in 22/42 patients, and angina scores were stable or improved in 41/42 patients. During treatment, no patients without left ventricular hypertrophy (LVH) at treatment initiation developed LVH, and no patients with LVH at treatment initiation showed a decline in left ventricular mass.

**Conclusions:**

Approximately 10 years of agalsidase alfa treatment appeared to have beneficial effects for controlling progression and improving some symptoms of Fabry-associated cardiomyopathy.

## Background

Fabry disease is a rare, inherited, X-chromosome linked glycosphingolipid storage disorder (OMIM 301500). In Fabry disease, mutations in the α-galactosidase A (GLA) gene cause functional deficiency of the enzyme α-GLA. In affected patients, lack of α-GLA activity leads to progressive accumulation of glycosphingolipids, particularly globotriaosylceramide (Gb_3_), in lysosomes, affecting almost all tissues and organs. By adulthood, patients with Fabry disease may experience a range of serious complications, such as cardiomyopathy, that result in significant morbidity and reduced life expectancy [1–10]. Because the GLA gene is located on the X-chromosome, males generally tend to be more severely affected than females, as the latter retain a physiologically significant level of enzyme α-GLA activity. Symptom onset in females, therefore, generally occurs later in life, with cardiomyopathy the most common and serious sign [9].

Cardiac changes in Fabry disease include progressive, infiltrative hypertrophic cardiomyopathy characterized by predominantly left ventricular (LV) wall thickening without cavity dilatation, structural changes in the mitral and aortic valves, and conduction abnormalities that may lead to increased susceptibility to arrhythmias [7–15]. The severity of cardiac symptoms correlates with overall disease progression over time, with intracellular deposition of Gb_3_ and fibrosis thought to be major, but not the only, mechanisms underlying these abnormalities [7, 8, 16–18].

Agalsidase alfa enzyme replacement therapy (ERT) for Fabry disease has been available for more than 10 years [19–23]. A number of studies of different designs support the efficacy and effectiveness of ERT, including agalsidase alfa, with regard to cardiac outcomes in adults [13, 21–27]. Reports of other studies, however, have suggested that ERT with either agalsidase alfa or beta may be less effective in patients with disease of longer duration and more severe symptoms at ERT initiation or that findings are equivocal [28–30].

To establish a clear understanding of the long-term effectiveness of agalsidase alfa in preventing and controlling the progression of cardiomyopathy in Fabry disease, we conducted a 10-year retrospective medical record review-based evaluation of morphological and functional cardiac changes in adult men and women treated with agalsidase alfa.

## Methods

### Study design and patients

A single-center retrospective analysis was carried out using prospectively collected data extracted from the medical records of patients with Fabry disease who were under the care of the University Children’s Hospital in Mainz, Germany, which is a participating center in the Fabry Outcome Survey (sponsored by Shire). Predefined data were extracted from medical records after written consent from patients and after local institutional review board and ethical committee approval of this analysis. Eligible patients had a Fabry disease diagnosis confirmed by enzyme assay (males) and/or DNA analysis (males and females), were aged ≥ 14 years at treatment start, and had received agalsidase alfa (Replagal®; Shire, Lexington, Massachusetts, USA) ERT for approximately 10 years.

All eligible patients received ERT continuously and none had ERT withdrawn or stopped. Female patients with intermittent reimbursement from the health care system were excluded from this analysis and 4 female patients were excluded because ERT was stopped for more than a year.

### Outcomes analyzed

Demographic characteristics and disease-related clinical parameters at the start of treatment were collected. Data were extracted for cardiac and heart failure status, echocardiographic evaluations of cardiac structure and function, and renal function at treatment start and then at predefined time points during agalsidase alfa treatment.

Heart failure and angina were evaluated as New York Heart Association (NYHA) classification of heart failure and Canadian Cardiovascular Society (CCS) grading of angina pectoris scores, respectively [30–32]. Detailed echocardiography was performed using digital echocardiographic equipment with appropriate transducers. Mean values were taken from instantaneous measurements made over 3 cardiac cycles from M-mode tracings according to American Society of Echocardiography recommendations [33]. Systolic and diastolic structural and functional parameters were calculated as described elsewhere [9, 14, 34]. LV mean wall thickness (MWT) measurements of ≥16 mm were considered to indicate severe LV hypertrophy (LVH) [35]. LV mass was calculated from echocardiographic measurement data and values indexed to patient height (LV mass index [LVMI]) [36]. Values of >50 g/m^2.7^ were considered indicative of LVH [35]. Systolic function was evaluated from LV ejection fraction (LVEF) measured using two-dimensional echocardiography [7, 9, 14, 37].

Renal function was evaluated from serum creatinine; estimated glomerular filtration rate (eGFR), calculated as eGFR Modification of Diet in Renal Disease values; [38] and time-averaged urinary protein excretion.

### Data analyses

Descriptive statistics were calculated for all values. Changes from baseline in study parameters were evaluated as changes in least squares (LS) mean values over time with 95 % confidence intervals (CI) adjusted for baseline demographic variables, or by calculating *t* statistic values. Differences were considered statically significant if p - values were ≤0.05. SAS/STAT® software version 9.2 (SAS Institute Inc., Cary, North Carolina, USA) was used for statistical analyses.

## Results

Forty-five patients (21 males, 24 females) met the criteria for inclusion in this analysis. Patients had been treated for a median (range) of 10.8 (9.6–12.5) years between January 2001 and December 2013. The mean (standard deviation [SD]) age at start of ERT was 38.7 (14.1) years for females and 30.2 (9.5) years for males. Mean blood pressure values and heart rates were within normal ranges (Table 1). One female and 2 male patients received pacemakers and 1 other male patient developed conduction abnormalities. Mean body mass index values were within healthy ranges at baseline (Table 1) but increased slightly over 10 years (LS mean [SD] change from baseline, males 1.74 [0.42] kg/m^2^; females 2.79 [0.48] kg/m^2^; both p < 0.0001). Virtually no residual enzyme activity was observed before treatment in male patients (median [range] 0.08 [0.00–0.14] units; n = 17), whereas female patients had activity indicative of heterozygosity (0.66 [0.35–1.09] units; n = 15). As previously described [25], the female patients all had signs or symptoms typical of Fabry disease (eg, neuropathic pain, gastrointestinal involvement, stroke, proteinuria, decreased renal function, and/or LVH) and had their Fabry diagnosis confirmed by mutation analysis.

**Table 1:** Demographic and basic clinical parameters at treatment start

Baseline parameter	Females	Males	Overall
N (%)	24 (53.3)	21 (46.7)	45
Age, mean (SD), years	38.6 (14.2)	30.2 (9.5)	34.7 (12.8)
Age at ERT start, mean (SD), years	38.7 (14.1)	30.2 (9.5)	34.7 (12.8)
Age at ERT start, median (range), years	37.2 (15.6–61.4)	31.4 (14.9–44.9)	34.5 (14.9–61.4)
BMI, mean (SD), kg/m^2^	23.5 (3.6)	21.6 (3.5)	22.6 (3.6)
Systolic BP, mean (SD), mmHg	127.3 (13.2)	126.2 (16.0)	126.8 (14.4)
Diastolic BP, mean (SD), mmHg	72.3 (9.1)	68.2 (10.3)	70.4 (9.8)
Heart rate, mean (SD), bpm	64.0 (13.1)	65.1 (7.7)	64.5 (10.8)
Diabetes, n (%)	0 (0)	0 (0)	0 (0)
Current smoker, n (%)	2 (8.3)	3 (14.3)	5 (11.1)
Arterial hypertension, n (%)	2 (8.3)	3 (14.3)	5 (11.1)

BMI: body mass index; bpm: beats per minute; BP: blood pressure; ERT: enzyme replacement therapy; SD: standard deviation

Concomitant therapies included: percutaneous transluminal coronary angioplasty in 2 patients; placement of a dual chamber pacemaker in 3 patients; dialysis in 1 patient who had progressed to end-stage renal disease; and beta-adrenergic blocking agents in 1 patient. In addition, all patients initiated therapy with angiotensin-converting enzyme inhibitors during the first 5 years of ERT. No patients received cardiac resynchronization therapy or an automatic implantable cardioverter defibrillator.

### NYHA classifications and CCS scores

**Fig. 1:**
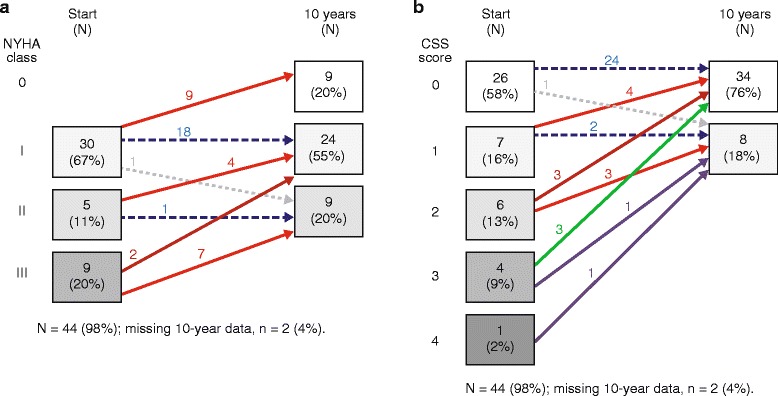
**a** Cluster analysis of NYHA heart failure classification. **b** Cluster analysis of CCS grading of angina pectoris scores before and after approximately 10 years of agalsidase alfa enzyme replacement therapy. Values in the boxes indicate the numbers (and percentages) of patients in that category. Arrows indicate directions of changes in classifications or scores, and numbers adjacent to arrows show the number of patients with that change. CCS: Canadian Cardiovascular Society; NYHA: New York Heart Association

At treatment start, heart failure symptoms (NYHA class ≥ II) were present in 31 % and anginal symptoms (CCS score ≥ 2) were present in 24 % of patients (Fig. 1). After 10 years of agalsidase alfa treatment, NYHA classification had improved by at least 1 class in 22/42 patients. Classifications were unchanged in 19 patients; only 1 patient had a worse classification, and no patients were classified as NYHA class III, compared with 9 before treatment (Fig. 1). Fifteen of 42 patients had an improved CCS score and 26 had a stable score after 10 years of ERT, with only 1 patient showing deterioration, and none having scores of 2, 3, or 4, compared with 11 patients before ERT (Fig. 1).

### Cardiac structural evaluations

Before ERT, mean (SD) MWT values for males (12.3 [2.9] mm; n = 21) and females (11.7 [2.5] mm; n = 24) indicated mild hypertrophy, although values ranged from normal to some instances of severe LVH (range, males 8.2–18.0 mm; females 8.7–17.0 mm). After 10 years of treatment, MWT was significantly reduced in males (LS mean [95 % CI] change −1.89 [−2.58, −1.19] mm; p < 0.0001), with changes apparent after 1 year (−2.08 [−2.69, −1.46] mm; p < 0.0001). Statistically significant changes were also apparent after 1 year in females (LS mean [95 % CI] change −2.01 [−2.55, −1.47] mm; p < 0.0001), although after 10 years, MWT was not significantly different from before treatment (−0.48 [−1.05, 0.09] mm; p = 0.0999).

**Fig. 2:**
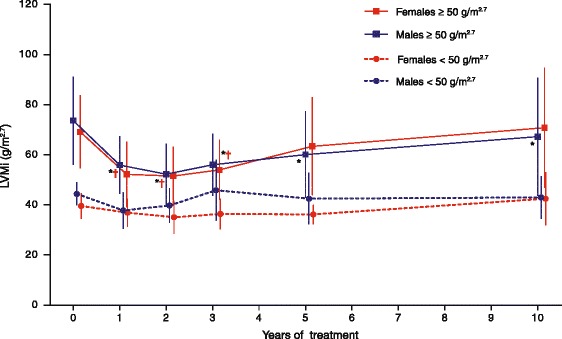
Left ventricular mass indexed to height during 10 years’ agalsidase alfa treatment for male and female patients stratified by LVMI <50 g/m^2.7^ or ≥50 g/m^2.7^ before treatment. Data points are means with standard deviations. LVMI: left ventricular mass index. *Statistically significant (p < 0.05) change from baseline among males with LVMI ≥50 g/m^2.7^ before treatment. †Statistically significant (p < 0.05) change from baseline among females with LVMI ≥50 g/m^2.7^ before treatment. ‡Statistically significant (p < 0.05) change from baseline among females with LVMI <50 g/m^2.7^ before treatment

At start of treatment, LVMI values suggested a varying degree of LVH, with 71 % (n = 15/21) of men and 67 % (n = 16/24) of women having LVMI ≥50 g/m^2.7^. After 10 years of ERT, LVMI was not significantly changed in patents with baseline LVMI <50 g/m^2.7^; however, in males with baseline values ≥50 g/m^2.7^, LVMI was significantly reduced after 10 years (LS mean [95 % CI] change −13.55 [−23.05, −4.06] g/m^2.7^; p = 0.0061; Fig. 2). A marked improvement was apparent in these patients after just 1 year (LS mean [95 % CI] change −16.46 [−23.81, −9.11] g/m^2.7^; p < 0.0001). A similar improvement after 1 year was observed in females with LVH and sustained for 3 years (1 year LS mean [95 % CI] change −16.69 [−23.62, −9.75] g/m^2.7^; p < 0.0001), although after 10 years, mean LVMI was found not to be significantly different from baseline (Fig. 2).

### Cardiac functional evaluations

Mean LVEF values before treatment were ≥60 % in all patients and were largely unchanged after 10 years of ERT in male patients (Table 2). In female patients, a statistically significant, albeit very slight, reduction in LVEF was observed; however, mean LVEF was still within the normal range. Mean (SD) heart rates remained similar to baseline after 10 years (males 67.4 [20.0] bpm; females 64.7 [11.7] bpm).

**Table 2:** Changes in left ventricular ejection fraction functional parameter, after approximately 10 years of agalsidase alfa treatment

	Females	Males
Left ventricular ejection fraction (%)		
Baseline, mean (SD)	71.9 (7.6), *n* = 23	69.9 (7.3), *n* = 21
10-year n, mean (SD)	68.4 (6.9)	69.8 (7.0)
Change from baseline^a^	−3.64 (−6.74, −0.54)	1.31 (−2.16, 4.78)
	*p* = 0.022	*p* =0.4546

^a^Adjusted changes from baseline: LS mean (95 % CI) changes

CI: confidence interval; LS: least squares; SD: standard deviation

### Renal function

**Fig. 3:**
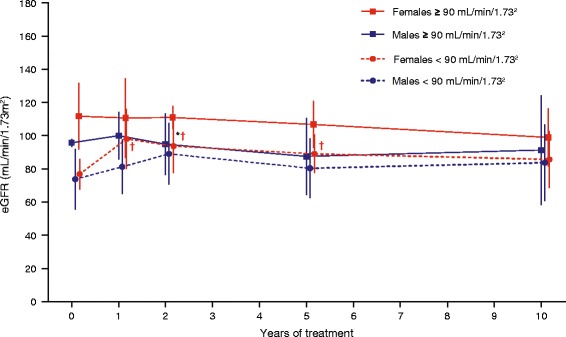
eGFR during 10 years’ agalsidase alfa treatment in male and female patients stratified by eGFR <90 or ≥90 mL/min/1.73 m^2^ before treatment. Data points are means with standard deviations. eGFR: glomerular filtration rate. *Statistically significant (p < 0.05) change from baseline among males with eGFR <90 mL/min/1.73 m^2^ before treatment. †Statistically significant (p < 0.05) change from baseline among females with eGFR <90 mL/min/1.73 m^2^ before treatment

Among patients (8 males, 5 females) with pretreatment eGFR ≥90 mL/min/1.73 m^2^, the mean annual decline in eGFR over 10 years was not statistically significant. In patients with poorer renal function (10 males, 11 females), eGFR seemed to improve in the first 3 years of ERT (Fig. 3). After 10 years, however, eGFR values were not significantly changed regardless of renal function before ERT. Serum creatinine values (baseline mean [SD] males 1.0 [0.2] mg/dL; n = 19; females 0.9 [0.2] mg/dL; n = 16) were also not significantly different after 10 years of ERT. Time-adjusted urinary protein levels did not change significantly over 10 years in male (mean [SD] 112.5 [35.8] mg/24 h; n = 8) and female patients (115.3 [48.0] mg/24 h; n = 6) without pre-treatment proteinuria (i.e. urine protein <200 mg/24 h at baseline), and in females with proteinuria (507.6 [388.9] mg/24 h; n = 7) at ERT initiation. However, in the 10 male patients who had proteinuria before treatment, urinary protein was significantly increased (baseline mean [SD] 659.0 [889.0] mg/24 h; 10-year change 297.0 [376.7] mg/24 h; p = 0.0342).

## Discussion

This study represents one of the largest and longest-term evaluations to date of the progression of cardiomyopathy in patients with Fabry disease who received 10 years of agalsidase alfa ERT.

The progression of untreated Fabry disease differs in some respects in men and women as a result of differences in levels of residual enzyme activity [3, 39–42]. Additionally, the differential cardiac effects in men and women may be caused by a mosaic distribution of affected and unaffected cardiac myocytes in women [2, 43]. Although symptoms appear approximately 10 years earlier in males, cardiac involvement is now understood to be prevalent in adult patients of both genders [2, 9, 15], with reports of ≥90 % of patients being affected by cardiomyopathy, with LVH in about half of untreated men and one third of untreated women, and >50 % of female patients reporting chest pain [13, 39–41]. The severity of cardiac abnormalities increases with age and, alongside renal disease, are a significant cause of morbidity and early mortality in both genders [2, 3, 7–10, 12, 13, 15, 39–41, 44]. A study of Fabry disease in 36 women (mean [SD] age, 47.0 [17.9] years) found that 25 (69 %) had LVH at baseline; after 4 years of agalsidase alfa ERT, there was significant reduction in LVMI in 22/25 (88 %) patients who had LVH at baseline, with 7/25 (28 %) no longer classified as having LVH [25]. Further, among those women with LVMI classified as normal at baseline, only 1 had progressed to LVH after 4 years of ERT [25].

One of the goals for ERT should be the prevention of future organ damage in patients who are not yet severely affected at the time of diagnosis, but who may develop symptoms later in their lives without treatment. It should also be noted that some patients will not develop progressive symptoms even without treatment and thus will have little or no benefit from treatment. The patient cohort presented here consists of classical Fabry patients with typical signs and symptoms of Fabry disease who still show excellent responses to treatment. However, despite the beneficial effects of ERT in patients with Fabry disease, patients in advanced stages of the disease may still die. From an analysis of deceased patients of our overall center cohort who have not been included in the analysis presented here due to their shorter treatment time (data not shown), no obvious overall demographic or Fabry disease-related differences were noted between those patients who died during the observation period and our patient cohort in total, except for the fact that more deceased patients were male and older at treatment initiation. Therefore, these non-responders may have been primarily those patients in whom ERT was initiated too late.

Our analysis included men and women with Fabry disease who had received agalsidase alfa for approximately 10 years. Improvements in NYHA heart failure classification and CCS angina scores were observed over this period. From a structural perspective, early improvements in MWT in male and female patients were observed, although there were no significant differences at 10 years. Also, LVMI values in the normal range before treatment were maintained over 10 years in men and women. Where LVH was present before ERT, improvements were apparent after just 1 year, with benefits in male patients sustained after 10 years and with deterioration controlled in females. Cardiac functionality parameters were stable over the 10-year study period, as evidenced by maintenance of LVEF values within the normal range. Electrocardiographic data are not presented because they do not reflect limited changes in LV mass and typically do not detect changes seen during ERT. Renal function was also generally maintained after 10 years of ERT. While differences are apparent in some of the 10-year analysis results between men and women, possibly because of the more severe effects of Fabry disease in men, we found that disease progression was generally attenuated and symptoms of cardiomyopathy were stable or improved in male and female patients who received 10 years of ERT.

A number of studies and analyses have explored the effects of agalsidase alfa on cardiomyopathy in Fabry disease over shorter time frames. Over treatment duration periods from 6 months to 5 years, agalsidase alfa has been reported to delay the onset of cardiac involvement, to reduce or stabilize LVMI in men and women with Fabry disease, and to reduce myocardial Gb_3_ in males [21–26]. Specifically, in patients with signs of LVH before ERT, decreases in LVMI over 1 to 5 years of treatment have been reported, whereas in patients without initial LVH, 3 to 5 years of treatment prevented progression [21, 23, 24]. Functionally, increased or stabilized mid-wall fractional shortening after 5 years of treatment regardless of baseline LVH has been reported [21], with no LVEF changes found in male patients after 6 months’ treatment in another study [22]. The findings of our retrospective analysis are thus in accord with those of previous studies of treatment over shorter time periods and further indicate the potential benefits of agalsidase alfa. Our results suggest that agalsidase alfa has long-term benefits for Fabry disease cardiomyopathy regardless of gender and of the severity of cardiac symptoms before treatment.

### Limitations

This was a retrospective analysis of data from medical records and not a prospective randomized controlled trial. Hence, patients were not randomly selected to receive ERT and the analysis did not include a comparator (control) group of untreated patients. Comparisons with pre-treatment data were therefore used to evaluate changes in parameters. As these baseline data were collected 10 years previously, the treated patients may have been considerably older when the comparisons were made, and aging itself may have had an adverse impact on the symptoms evaluated. Although M-mode echocardiography has inherent limitations of variability and reproducibility, and thus is not the ideal method for serial measurement of LVMI, it is widely available and commonly used. Finally, patients whose data were included had been referred for specialist treatment and, in the case of the female patients, severe disease was more likely.

## Conclusions

In this 10-year evaluation of adult men and women with Fabry disease who received agalsidase alfa treatment, there was no progression of cardiomyopathy as determined by a range of structural and functional parameters.

## Abbreviations

LVH: Left ventricular hypertrophy
LV: Left ventricular
ERT: Enzyme replacement therapy
NYHA: New York Heart Association
CCS: Canadian Cardiovascular Society
MWT: Mean wall thickness
LVEF: LV ejection fraction
eGFR: estimated glomerular filtration rate
LS: Least squares
LVMI: Left ventricular mass index
