# Erratum to: Effectiveness of agalsidase alfa enzyme replacement in Fabry disease: cardiac outcomes after 10 years’ treatment

**DOI:** 10.1186/s13023-016-0482-3

**Published:** 2016-07-12

**Authors:** Christoph Kampmann, Amandine Perrin, Michael Beck

**Affiliations:** Section Head for Congenital Heart Defects, Center for Pediatric and Adolescent Medicine, University Medical Center, University of Mainz, Langenbeckstr. 1, Mainz, DE-55101 Germany; Statistical Programmer, Rare Diseases Business Unit, Global Outcomes Research, Shire, Zug, Switzerland; Professor Emeritus, Department of Pediatrics, University Medical Center, University of Mainz, Mainz, Germany

## Erratum

Following the publication of our article “Effectiveness of agalsidase alfa enzyme replacement in Fabry disease: cardiac outcomes after 10 years’ treatment” by Kampmann et al. [1] we have become aware that the dose of agalsidase alfa was not reported.

Eligible patients had a Fabry disease diagnosis confirmed by enzyme assay (males) and/or DNA analysis (males and females), were aged ≥14 years at treatment start, and had received agalsidase alfa (Replagal®; Shire, Lexington, Massachusetts, USA) ERT at a dose of 0.2 mg/kg body weight every other week for approximately 10 years.
